# Lung ultrasound in seven children in a Pediatric Intensive Care Unit- comparison among chest X ray, chest CT and lung ultrasound

**DOI:** 10.1186/2036-7902-7-S1-A9

**Published:** 2015-03-09

**Authors:** S Fukuhara, Y Ishida, S Tsuji, M Kusumoto, S Kajihara, Y Yamaguchi, H Takeda, Y Uetani

**Affiliations:** Department Of Emergency And Critical Care Medicine, Kobe Children's Hospital, Kobe City, Japan

**Keywords:** Pneumonia, Lung Disease, Respiratory Failure, Pleural Effusion, Pulmonary Edema

## Background

Respiratory failure is one of the most common and critical problems in pediatric intensive care units (PICUs). The accurate precise assessment of respiratory failure and precise diagnoses of lung diseases are key issues in PICUs. Assessments by chest X rays (CXR) are common and prevalent for determining the reasons for respiratory failure in children. However, CXRs can be misread. Some patients may require chest Computed Tomography (CCT). CCT is essential for finding the abnormal region. However, in the PICU, the number of children who need CCT is small, and the risk of transporting unstable patients and the possibilities of malignancies are problematic. Lung ultrasound (LUS) has proven useful for detecting lung abnormalities in adults, but its usefulness is not clear in children.

## Objective

Comparison among CXR, CCT, and LUS in children in a PICU.

## Patients and methods

We present a series of seven children who were admitted to a 10-bed PICU in a tertiary children’s hospital in Japan. Each child underwent CXR, CCT, and LUS.

## Results

The image findings in 2 cases who had interstitial pneumonia or pulmonary edema corresponded between CCT and LUS, but in a case with possible mild interstitial pneumonia with mild symptoms, we were not able to detect the B-lines on LUS. In each case, the use of CXR was impossible for evaluating pneumonia, and there were no obvious abnormalities. Atelectasis, pneumonia, and pleural effusion were detectable both with CCT and LUS. Some CXRs were able to detect abnormalities in the lung regions, but qualitative assessments were difficult in some cases, such as those with atelectasis or pleural effusion that was comorbid with substantial pneumonia.

## Conclusion

In our experience with seven cases, LUS was safe and useful. We believe that LUS can be beneficial for evaluating children in the PICU who have respiratory failure. LUS is safe and available in PICUs.
